# Monitoring the elimination of human African trypanosomiasis at continental and country level: Update to 2018

**DOI:** 10.1371/journal.pntd.0008261

**Published:** 2020-05-21

**Authors:** José R. Franco, Giuliano Cecchi, Gerardo Priotto, Massimo Paone, Abdoulaye Diarra, Lise Grout, Pere P. Simarro, Weining Zhao, Daniel Argaw

**Affiliations:** 1 World Health Organization, Control of Neglected Tropical Diseases, Geneva, Switzerland; 2 Food and Agriculture Organization of the United Nations, Animal Production and Health Division, Rome, Italy; 3 World Health Organization, Regional Office for Africa, Communicable Disease Unit, Brazzaville, Congo; 4 Consultant World Health Organization, Control of Neglected Tropical Diseases, Geneva, Switzerland; Makerere University, UGANDA

## Abstract

**Background:**

In 2012 human African trypanosomiasis (HAT), also known as sleeping sickness, was targeted for elimination as a public health problem, set to be achieved by 2020. The World Health Organization (WHO) provides here the 2018 update on the progress made toward that objective. Global indicators are reviewed, in particular the number of reported cases and the areas at risk. Recently developed indicators for the validation of HAT elimination at the national level are also presented.

**Methodology/Principal Findings:**

With 977 cases reported in 2018, down from 2,164 in 2016, the main global indicator of elimination is already well within the 2020 target (i.e. 2,000 cases). Areas at moderate or higher risk (i.e. ≥ 1 case/10,000 people/year) are also steadily shrinking (less than 200,000 km^2^ in the period 2014–2018), thus nearing the 2020 target [i.e. 90% reduction (638,000 km^2^) from the 2000–2004 baseline (709,000 km^2^)]. Health facilities providing diagnosis and treatment of gambiense HAT continued to increase (+7% since the previous survey), with a better coverage of at-risk populations. By contrast, rhodesiense HAT health facilities decreased in number (-10.5%) and coverage. At the national level, eight countries meet the requirements to request validation of gambiense HAT elimination as a public health problem (i.e. Benin, Burkina Faso, Cameroon, Côte d’Ivoire, Ghana, Mali, Rwanda, and Togo), while for other endemic countries more efforts are needed in surveillance, control, or both.

**Conclusions/Significance:**

The 2020 goal of HAT elimination as a public health problem is within grasp, and eligible countries are encouraged to request validation of their elimination status. Beyond 2020, the HAT community must gear up for the elimination of gambiense HAT transmission (2030 goal), by preparing for both the expected challenges (e.g. funding, coordination, integration of HAT control into regular health systems, development of more adapted tools, cryptic trypanosome reservoirs, etc.) and the unexpected ones.

## Author summary

Human African trypanosomiasis (HAT), a lethal disease transmitted by tsetse flies, wreaked havoc in Africa at different times in the 20^th^ century. Over the past twenty years, huge efforts made by a broad coalition of stakeholders curbed the last epidemic and brought the disease to the brink of elimination. In this paper, the latest figures on disease occurrence, geographical distribution and control activities are presented. Strong evidence indicates that the elimination of sleeping sickness ‘as a public health problem’ by 2020 is well within reach. In particular, fewer than one thousand new cases were reported in 2018, and the area where the risk of infection is estimated as moderate, high or very high has shrunk to less than 200,000 km^2^. More than half of this area is in the Democratic Republic of the Congo. The interruption of transmission of the gambiense form, targeted by the World Health Organization (WHO) for 2030, will require renewed efforts to tackle a range of expected and unexpected challenges. The rhodesiense form of the disease represents a small part of the overall HAT burden. For this form, the problem of under detection is on the rise and, because of an important animal reservoir, the elimination of disease transmission is not envisioned at this stage.

## Introduction

Human African trypanosomiasis (HAT, also known as sleeping sickness) is a parasitic infection transmitted by tsetse flies (Genus: *Glossina*). *Trypanosoma brucei gambiense* is the agent of the slow-progressing form that occurs in western and central Africa, while *T*. *b*. *rhodesiense* is found in eastern and southern Africa where it causes a faster-progressing form [1, 2].

Following a serious recrudescence in the late 1990s [3], strengthened control and surveillance activities over the past twenty years succeeded in progressively reducing disease transmission [4, 5]. National Sleeping Sickness Control Programmes (NSSCPs) have been the front-line actors in this success story, with the support of the World Health Organization (WHO), its public-private partnership with pharmaceutical companies Sanofi and Bayer, and a range of other partners and stakeholders, including bilateral cooperation agencies, non-governmental organizations (NGOs) and philanthropic organizations.

Progress in disease control was such that WHO, in its 2012 Neglected Tropical Diseases (NTD) roadmap [6], targeted the elimination of sleeping sickness as a public health problem for 2020. Beyond that, WHO and disease endemic countries targeted the elimination of transmission of gambiense HAT, resulting in zero cases reported, by 2030. Because of the zoonotic nature of rhodesiense HAT and the limited availability of control tools, the elimination of transmission of this form of the disease is presently considered not feasible [7, 8].

Global indicators and milestones to monitor the progress towards the 2020 goal of HAT elimination as a public health problem were defined and endorsed by various WHO experts groups, including an ‘Expert Committee’ on HAT [4], the ‘HAT elimination Technical Advisory Group’ (HAT-e-TAG), and the ‘Neglected Tropical Diseases Scientific and Technical Advisory Group’ (NTD-STAG). Based on these global indicators, updates on the progress towards HAT elimination are published every two years [5, 9, 10] and presented at various WHO HAT stakeholders meetings [7, 11–13]. The present paper provides the update to 2018.

In addition to the global picture, the progress towards HAT elimination needs to be followed at the national level as well. In particular, in its ‘Generic framework for control, elimination and eradication of NTDs’, WHO defines ‘validation’ as the process of documenting ‘elimination as a public health problem’, and ‘verification’ as the process of documenting ‘elimination of transmission’ [14]. For WHO to acknowledge the claims made by Member States in relation to their ‘validation’ or ‘verification’ status, disease-specific dossiers need to be submitted to WHO and to be assessed by a reviewing authority [14].

The available global metrics for HAT elimination cannot be readily applied for the validation at the national level. Because of this, the WHO HAT-e-TAG developed country-level indicators that can be easily measured by endemic countries and used in the validation dossiers. These new indicators and the related targets are published in this paper for the first time, alongside a preliminary assessment of the eligibility status of HAT-endemic countries.

## Materials and Methods

### Ethics statement

This paper does not involve research with human participants. No individual data is used. All the data used are provided routinely by NSSCPs, NGOs and other institutions collecting epidemiological information and are fully anonymized before transmission.

### Global indicators of HAT elimination as a public health problem

At the global level, two primary indicators are used to monitor the progress towards the 2020 goal of HAT elimination as a public health problem: (1) the number of cases reported annually (target: fewer than 2,000); and (2) the area at risk reporting ≥ 1 case/10,000 people/year, calculated over 5-year periods [target: a reduction of 90% (i.e. 638,000 km^2^) by 2016–2020 compared to the 2000–2004 baseline (i.e. 709,000 km^2^)] [4, 15, 16]. Although not linked to specific targets, three secondary indicators are also monitored: (a) the disease geographic distribution, (b) the at-risk population, and (c) the coverage of the at-risk population by control and surveillance activities [4].

The epidemiological data to calculate all indicators are provided by the NSSCPs, NGOs and research institutions, and systematically collated in the Atlas of HAT [17]. Earlier updates on the progress towards elimination were given for 2012 [9], 2014 [10] and 2016 [5], whilst previous dedicated publications dealt with the methodological details, not repeated here for the sake of brevity[17–22].

In order to update the number and distribution of fixed health facilities with capacity for HAT diagnosis and treatment, as well as their specific capacities, a comprehensive survey is carried out every two years. These data are used to estimate the coverage of the at-risk population [22].

### Indicator of HAT elimination as a public health problem at country level

The HAT-e-TAG defined HAT elimination as a public health problem at country level as the situation where fewer than 1 case/10,000 inhabitants/year (averaged over a 5-year period), are reported in each health district of the country, in conjunction with adequate, functional control and surveillance. In particular, information on the intensity and effectiveness of HAT control and surveillance activities is needed, including active and passive case finding, vector control and African animal trypanosomiasis (AAT) control. The indicators are applicable to both gambiense and rhodesiense HAT.

As a guide to eligibility for validation of elimination, the overall intensity and effectiveness of the HAT control and surveillance activities at the national level is assessed and ranked into three categories: ‘adequate’, ‘insufficient’ or ‘absent’.

In the present paper, a preliminary assessment of the countries’ validation status was carried out by WHO and NSSCPs coordinators. Data from the Atlas of HAT for the years 2014–2018 were used to estimate the main indicator, whilst information provided by, and consultations with NSSCP’s enabled an assessment of the control and surveillance activities.

## Results

### Number of HAT cases reported annually

**Fig 1:**
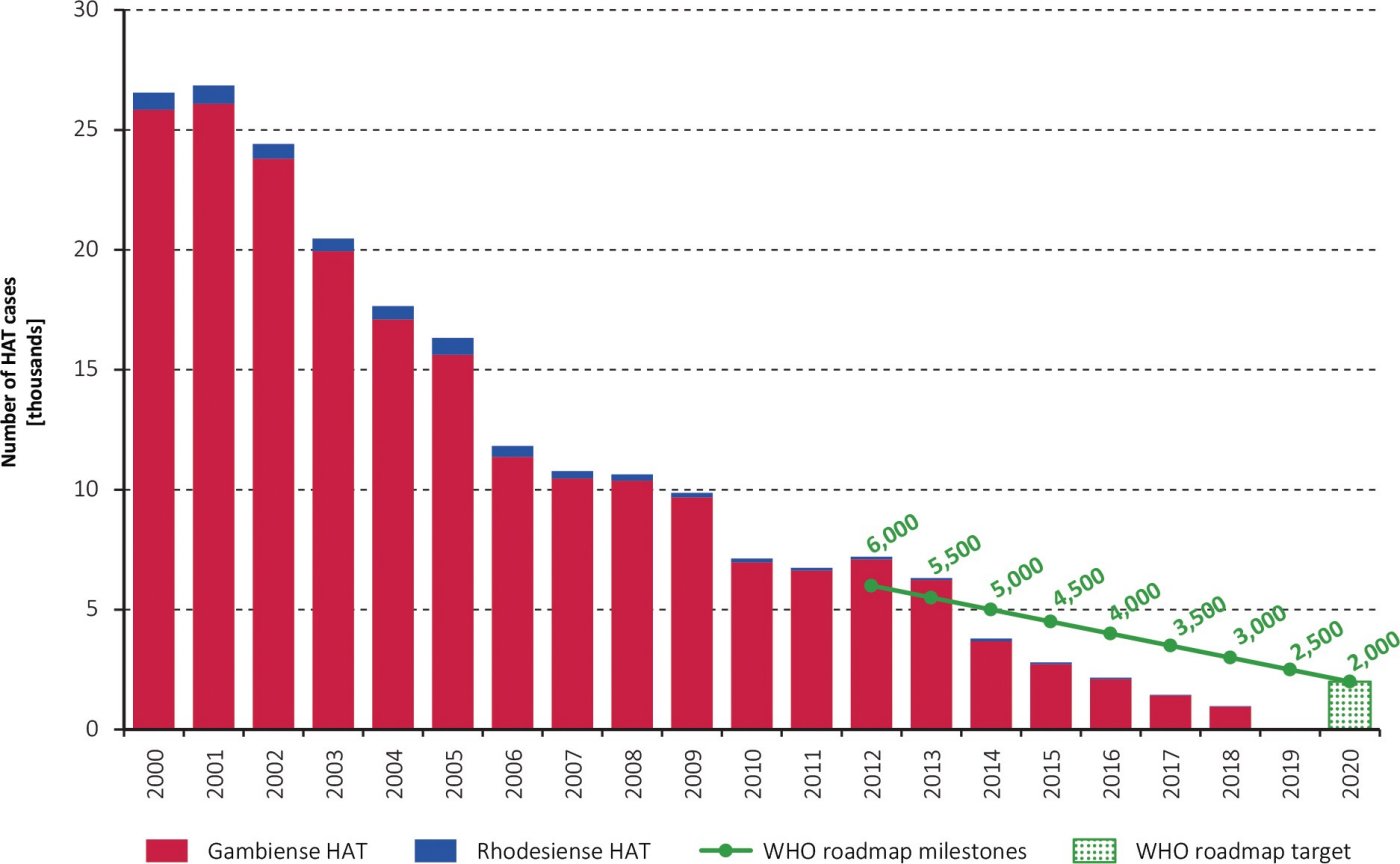
Total number of reported cases of HAT (gambiense and rhodesiense) per year (2000–2018). The green line and the green bar show the milestones and target set in the WHO Roadmap for HAT elimination [6].

HAT cases reported by country in the past ten years (period 2009–2018) are shown in Table 1 (*T*. *b*. *gambiense*) and Table 2 (*T*. *b*. *rhodesiense*). With a total of 977 cases reported in 2018 (including both gambiense and rhodesiense HAT), and 1,436 reported in 2017, the first global indicator of HAT elimination as a public health problem has already been achieved (Fig 1).

**Table 1:** *T*. *b*. *gambiense* HAT: New cases reported between 2009 and 2018.

Country	2009	2010	2011	2012	2013	2014	2015	2016	2017	2018	Total
Angola	247	211	154	70	69	36	35	20	18	79	939
Burkina Faso	0	0	0	0	0	0	1	0	0	0	1
Cameroon	24	16	15	7	6	7	6	6	5	7	99
Central African Republic	1,054	395	132	381	59	194	147	101	76	57	2,596
Chad	510	232	276	197	195	95	67	54	28	12	1,666
Congo	87	87	61	39	20	21	36	18	15	24	408
Côte d’Ivoire	8	8	10	9	7	6	3	0	3	2	56
Democratic Republic of the Congo	7,178	5,624	5,590	5,969	5,649	3,205	2,347	1,768	1,100	660	39,090
Equatorial Guinea	7	8	1	2	3	0	0	3	4	4	32
Gabon	14	22	17	9	17	10	9	10	9	16	133
Ghana	0	0	0	0	1	0	0	0	0	0	1
Guinea	79	68	57	70	78	33	29	108	139	74	735
Nigeria	0	2	3	2	0	0	0	1	0	0	8
South Sudan	373	199	272	317	117	63	45	17	12	17	1,432
Uganda	99	101	44	20	9	9	4	4	0	1	291
Total	9,680	6,973	6,632	7,092	6,230	3,679	2,729	2,110	1,409	953	47,487

Other historically *T*. *b*. *gambiense* HAT endemic countries not reporting cases but with surveillance activities are Benin, Mali, Guinea-Bissau, Niger, Senegal, Sierra Leone, and Togo. In the Gambia and Liberia no cases are reported but no surveillance activity is known.

**Table 2:** *T*. *b*. *rhodesiense* HAT: New cases reported between 2009 and 2018.

Country	2009	2010	2011	2012	2013	2014	2015	2016	2017	2018	Total
Kenya	1	0	0	2	0	0	0	0	0	0	3
Malawi	39	29	23	18	35	32	30	35	7	15	263
Uganda	129	112	84	71	43	70	28	10	13	4	564
United Republic of Tanzania	14	5	1	4	2	1	2	4	3	0	36
Zambia	4	8	3	6	6	12	9	4	3	5	60
Zimbabwe	3	2	4	9	1	3	3	1	1	0	27
Total	190	156	115	110	87	118	72	54	27	24	953

Other historically *T*. *b*. *rhodesiense* HAT endemic countries not reporting cases are Burundi, Ethiopia, Mozambique and Rwanda. Botswana, Namibia and Eswatini are considered free of the vector for the transmission of *T*. *b*. *rhodesiense* HAT [23–26].

Cases of gambiense HAT represent the main burden of the disease (i.e. 98% of the total), but compared to 2000 their number decreased by 96%. In the last two years of reporting (2017–2018), the Democratic Republic of the Congo accounted for 74% of the gambiense HAT cases. At continental level, the intensity of active case finding has been maintained at the same level as previous years, with 2.3 and 2.7 million people screened in 2017 and 2018 respectively (average for the period 2000–2016: 2.4 million). In turn, the number of people screened per case detected continues to increase, as in 2018, 5,455 people needed to be screened per each gambiense HAT case actively detected (i.e. 2,694,593 people screened / 494 cases actively detected).

Concerning rhodesiense HAT, 27 and 24 cases were reported in 2017 and 2018 respectively, which constitute 2% of the total HAT reported cases. The figure in 2018 represents a reduction of 97% compared to the year 2000.

#### Human African trypanosomiasis cases detected in non-endemic countries

HAT cases are occasionally diagnosed in non-endemic countries, most frequently in travellers and migrants [21]. In the past 10 years, such cases have been reported to WHO at an average of 6 per year, and two thirds of them were of the rhodesiense form (Table 3). Information on these cases is regularly transmitted from WHO to the NSSCP in the endemic countries, and they are included in the total tally of reported cases (Table 1 and Table 2).

**Table 3:** HAT cases diagnosed in non-endemic countries and reported to WHO (2009–2018).

Country	2009	2010	2011	2012	2013	2014	2015	2016	2017	2018	Total
Rhodesiense HAT	9	6	1	8	2	2	4	4	3	4	43
Gambiense HAT	4	4	0	2	3	1	0	3	1	1	19
Total	13	10	1	10	5	3	4	7	4	5	62

Focusing on the biennium 2017–2018, 7 cases of rhodesiense HAT and 2 cases of gambiense HAT were reported from non-endemic countries. They were detected in China (2), Netherlands (2), South Africa (2), France (1), Germany (1) and India (1). The country of origin, as well as the probable place of infection, are determined from the patient’s epidemiological history. For these 9 cases, infections were estimated to have occurred in Malawi (2), United Republic of Tanzania (2), Zambia (2), Gabon (1), Guinea (1) and Uganda (1).

### Geographic distribution of HAT

**Fig 2:**
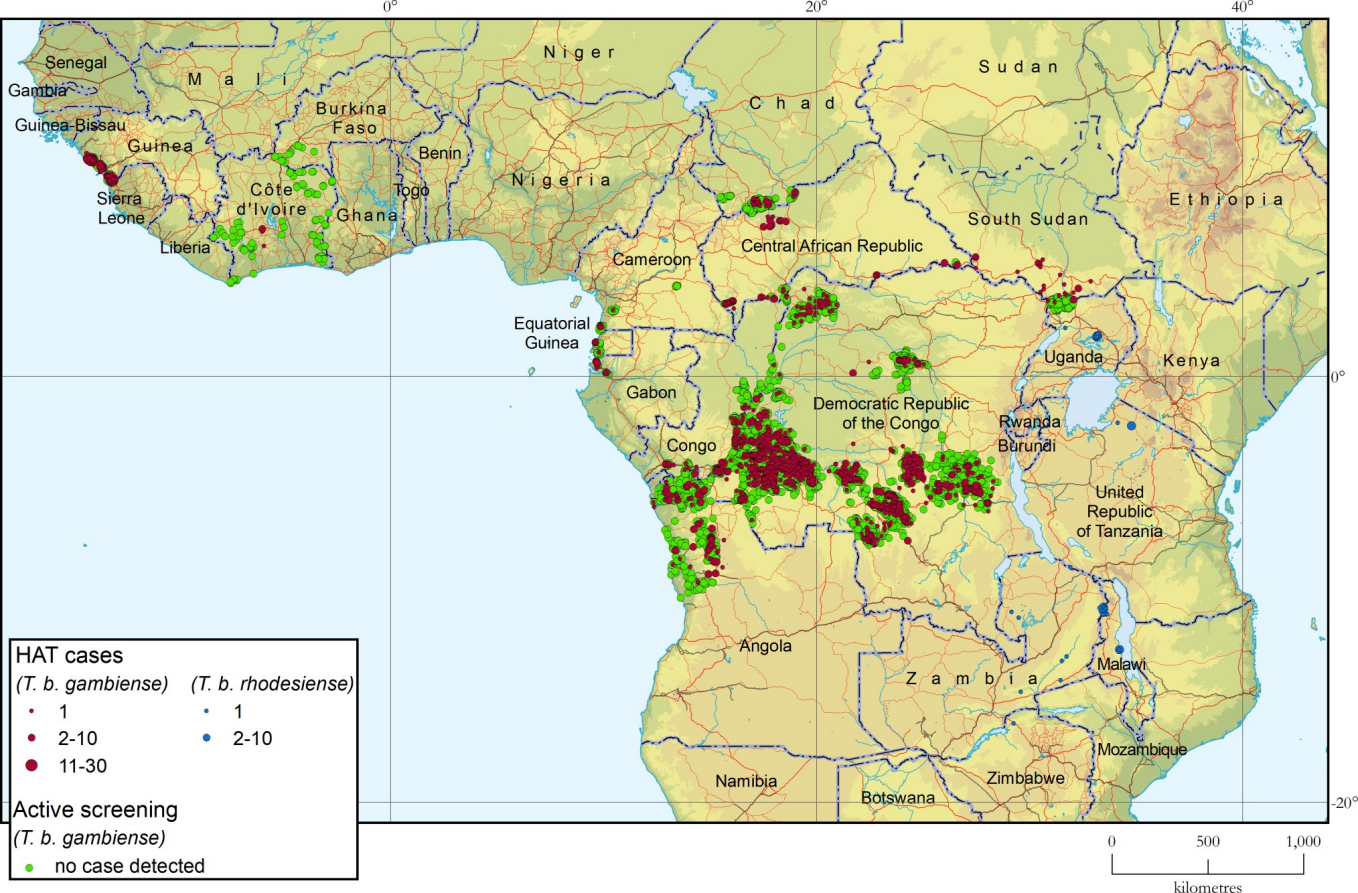
Geographic distribution of human African trypanosomiasis. Period 2017–2018.

Fig 2 shows the geographic distribution of sleeping sickness cases for the 2-year period 2017–2018. The locations of active screening activities where no cases were detected are included (green dots).

#### Gambiense HAT

In West Africa, cases are reported mainly from Guinea and, to a smaller extent, Côte d’Ivoire. In Guinea, following a serious lapse during the 2014–2015 Ebola epidemic, control and surveillance activities were resumed, with a resulting spike in reported cases in 2016 and 2017. In 2018 reported cases fell back to pre-Ebola levels. In Côte d’Ivoire, sporadic cases continue to be reported against a backdrop of reinforced case detection activities. No other country in the region reported cases in the period 2017–2018. Benin, Burkina Faso, Ghana, Mali and Togo have maintained the operational level of HAT surveillance, while in Nigeria a weakening has been observed. During 2017 and 2018, no surveillance and control activities were carried out in the Gambia, Guinea-Bissau, Liberia, Senegal, Sierra Leone and Niger.

In Central Africa, HAT control and surveillance activities are routinely carried out in Cameroon, Chad, Congo, Equatorial Guinea and Gabon. In Cameroon and Equatorial Guinea, the number of reported cases is stable at the single-digit level of previous years. In Chad and Central African Republic (CAR) a significant decrease was observed, with a 78% (Chad) and 44% (CAR) reduction in reported cases between 2016 and 2018. In Chad, control activities were maintained at an adequate level, with the addition in 2014 of a vector control programme that greatly reduced the tsetse population in the Mandoul focus [27]. In CAR, despite some improvement in the security situation, certain endemic areas are still not accessible and a sizable portion of the population remains displaced. By contrast, in Congo and Gabon an increase in the number of reported cases was observed. This trend is a reason for concern, even though it has to be interpreted in the context of reinforced passive surveillance in Congo and slightly increased active screening activities in Gabon.

In Uganda, a single autochthonous case of gambiense HAT was reported in the period 2017–2018, thus confirming previous positive trends and the substantial progress made towards disease elimination [28].

In South Sudan, the number of reported cases remains low, but it has to be considered that the persistent civil strife and a weak health system greatly hamper control and surveillance activities.

In Angola, the number of cases doubled in 2017–2018, compared to 2015–2016. In particular, 79 cases were detected in 2018, a level not reached since 2011. This spike owes to the reinforcement of control and surveillance activities; in particular, the coverage of passive screening was broadened, its targeting was improved, and active screening activities were scaled up.

The Democratic Republic of the Congo is still the country with the heaviest burden of gambiense HAT, but the number of cases continues in its steady downward trend. As compared to 2016 (1,768 cases), 2018 saw a two-third reduction (660 cases reported). The decrease is mainly ascribed to sustained control efforts in most endemic areas. However, insufficient control and surveillance activities still characterize some areas (e.g. Haut Uele Province, in the north-eastern part of the country).

#### Rhodesiense HAT

The number of reported cases of rhodesiense HAT has reached very low levels (27 cases in 2017 and 24 in 2018). This is mainly linked to a reduction in Malawi and Uganda, which remain the countries with the highest burden. Tanzania, Zambia and Zimbabwe continue to report sporadic cases, mainly related to the wildlife reservoir. Rwanda, backed-up by an adequate surveillance system, does not report cases. Kenya reports no cases but the level of surveillance has been reduced in recent years.

A general weakening in the capacities of diagnosis of rhodesiense HAT was observed, which raises concerns about the true epidemiological situation underlying the reported trends. Reports of rhodesiense HAT cases diagnosed in non-endemic countries among travellers, remain relatively numerous (i.e. two in South Africa, two in the Netherlands, two in China and one each in France, Germany and India for the period 2017–2018), and they account for 18% of the total reported cases (i.e. 9/51). Such a ratio is suggestive of a non-negligible level of under detection in rhodesiense HAT endemic countries.

### Areas and population at risk of HAT

#### Areas at risk of HAT

**Fig 3:**
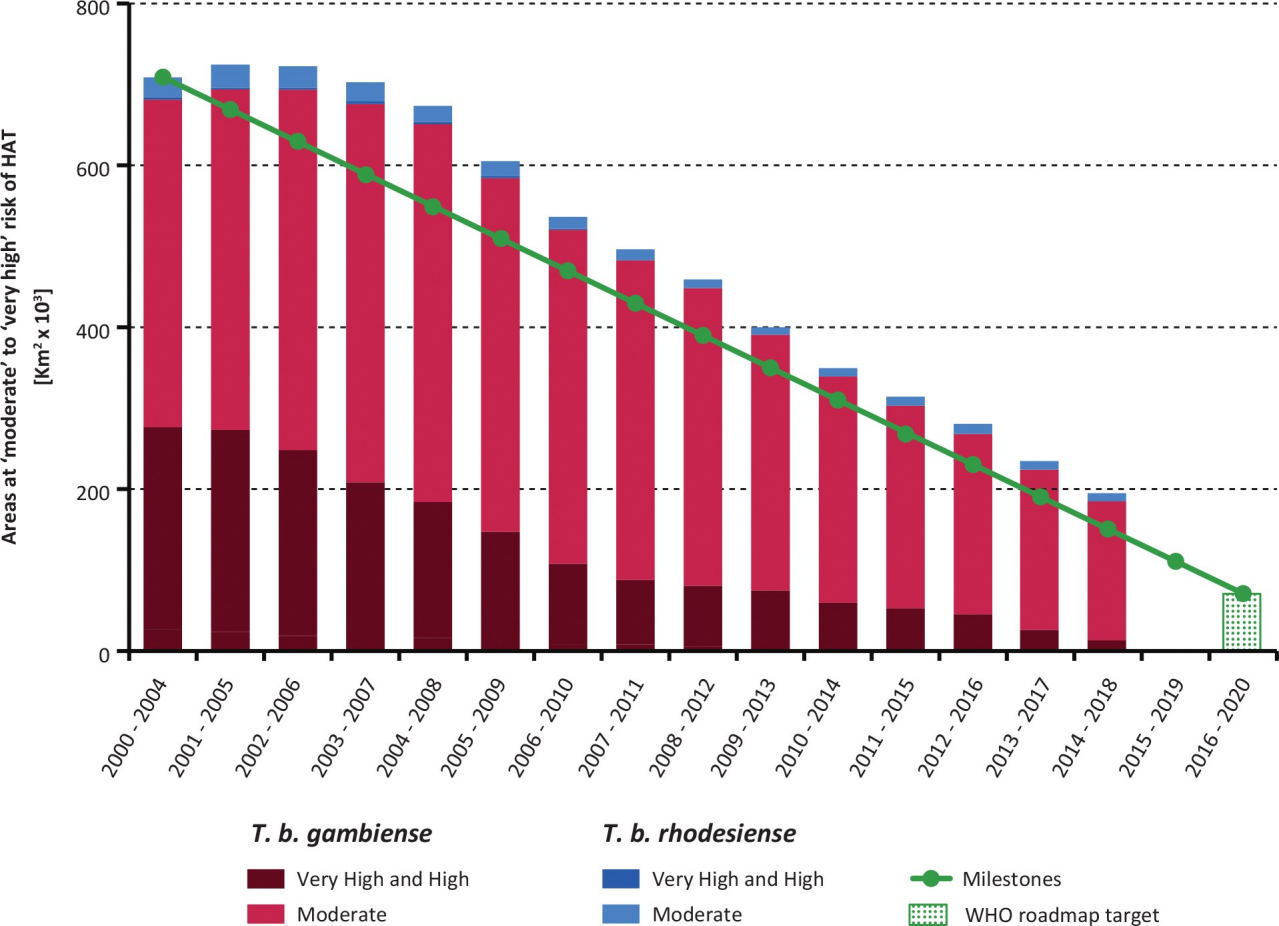
Trends in area at risk of gambiense and rhodesiense HAT where the disease is still considered as a public health problem (2000–2004 to 2014–2018). The green line shows the milestones set by the WHO-NTD-STAG to achieve the elimination of HAT as a public health problem by 2020.

**Fig 4:**
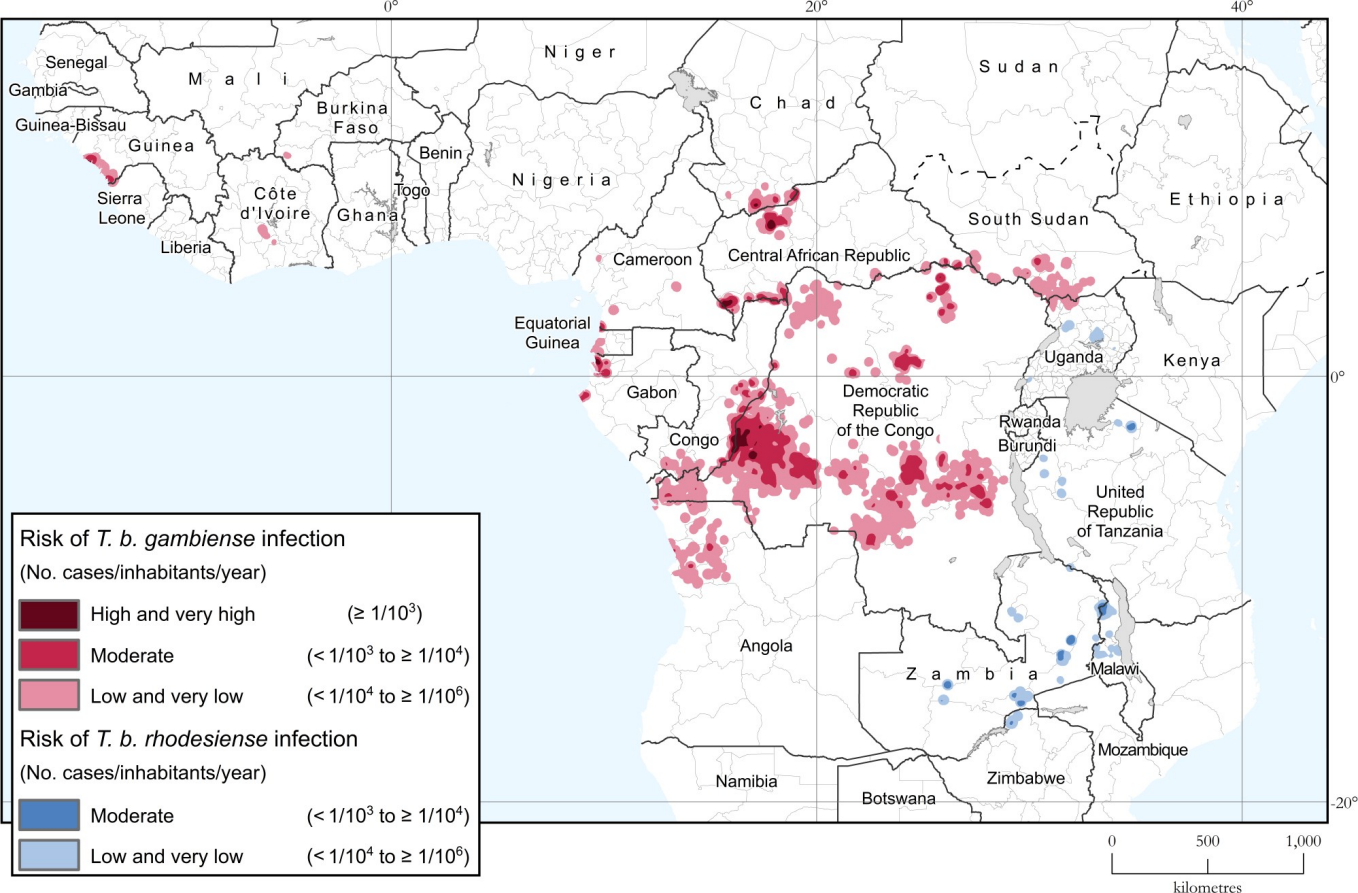
Areas at risk of HAT infection. Period 2014–2018.

In 2017 and 2018 the areas at very high, high or moderate risk for HAT continued the steady decline observed in the last ten years (i.e. a reduction of over 40,000 km^2^/year) (Fig 3). Overall, these areas reporting ≥ 1 case/10,000 inhabitants/year now stretch over 195,000 km^2^ (period 2014–2018). Although this value is still 29% higher than the corresponding elimination milestone (i.e. 151,000 km^2^), the rate of the decline is such that the ambitious 2020 target seems within reach (i.e. a reduction of 90% from the 2000–2004 baseline of 709,000 km^2^). Fig 4 shows the geographic distribution of the areas at risk for the period 2014–2018 (including the area at low and very low risk), while S1 File provides the country breakdown.

Looking at gambiense HAT, an overall 866,000 km^2^ are estimated to be at risk. Only 1.5% of this area (i.e. 13,000 km^2^) is at high or very high risk. The area at moderate risk is 171,000 km^2^ (i.e. 20% of the total risk area). Sixty-five percent of the area at gambiense HAT risk is in the Democratic Republic of the Congo (i.e. 566,000 km^2^, or a quarter of the total country area), followed by Angola (8.5%) and Central African Republic, Congo and South Sudan (6% each).

For rhodesiense HAT, approximately 71,000 km^2^ are estimated to be at risk, with no area at high or very high risk, and only 10,000 km^2^ at moderate risk. Zambia is the country with the largest area at risk (32,000 km^2^), followed by Malawi (14,000 km^2^) and Tanzania (12,000 km^2^).

#### Population at risk of HAT

For the period 2014–2018 less than 54 million people are estimated to be at various levels of risk of sleeping sickness, of which approximately 11% are at moderate risk or higher. The corresponding country breakdown is in S2 File.

Gambiense HAT is responsible for 95% of the people at risk (i.e. 51 million), of which 10.8% are at moderate risk and less than 0.3% are at high or very high risk. Thirty-seven million people are at various levels of risk in the Democratic Republic of the Congo (44% of the country population), followed by Guinea (3 million), Congo (2.5 million) and Angola and South Sudan (2 million each).

Less than 3 million people are estimated to be at risk of rhodesiense HAT, 97% of which are in the low and very low risk categories. The largest at-risk population is found in Uganda and Malawi (1 million people each).

### Population at risk potentially covered by fixed health facilities with capacities for HAT diagnosis and treatment

#### Survey and mapping of fixed health facilities

**Fig 5:**
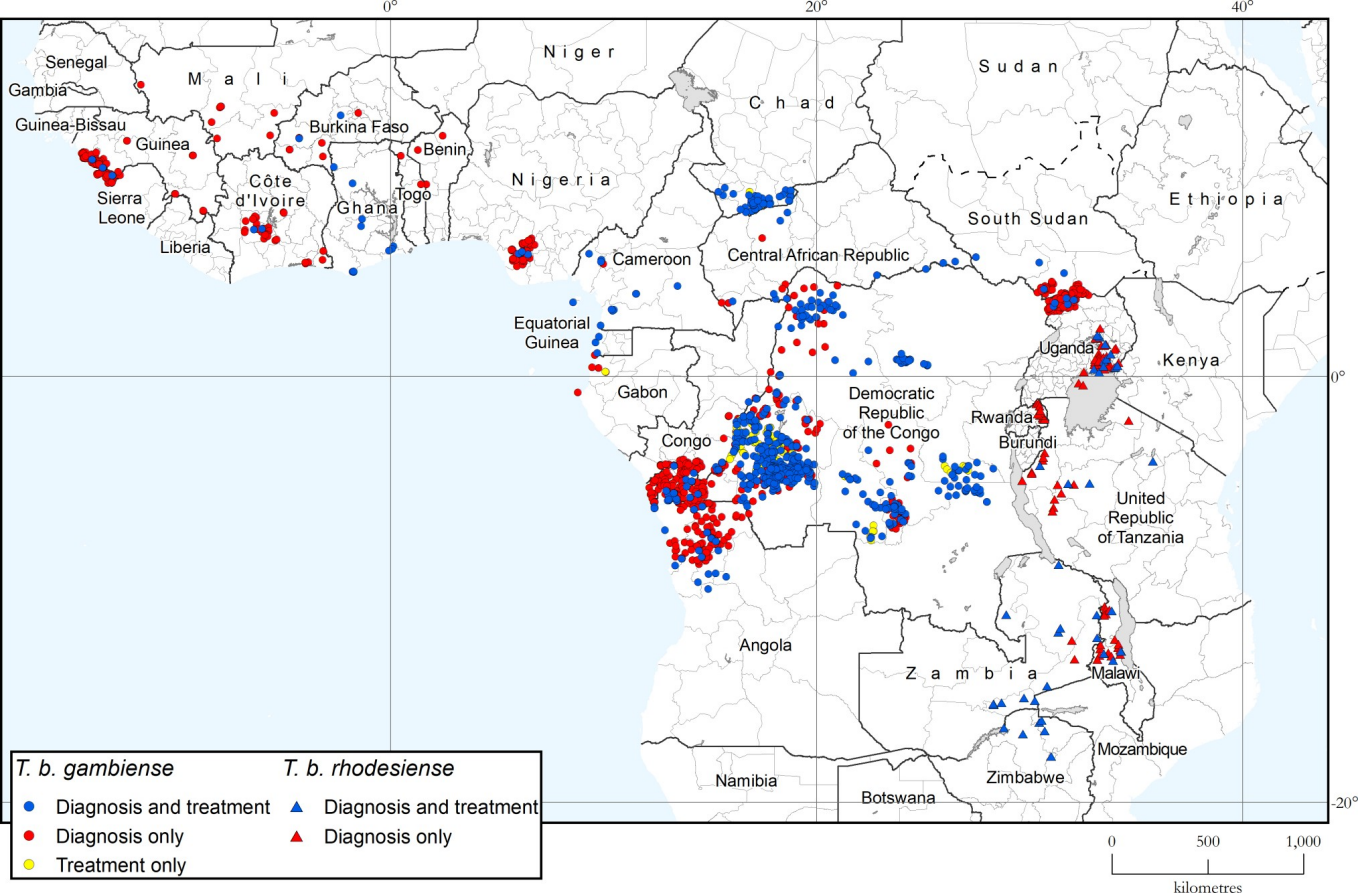
Geographic distribution of fixed health facilities offering diagnosis and treatment of gambiense and rhodesiense HAT. Data were collected by WHO from National Sleeping Sickness Control Programmes in July 2019

Table 4 and Table 5 show the fixed health facilities with capacity for diagnosis or treatment of gambiense and rhodesiense HAT respectively. The data result from the survey completed in July 2019, and the geographic distribution of the facilities is shown in Fig 5.

**Table 4:** Fixed health facilities for gambiense HAT: July 2019 survey. Differences between the July 2019 survey and the June 2017 survey [2] in column ‘Δ’.

Country	Diagnosis						Treatment						TOTAL	Δ
	DxC	DxS	DxP	DxPh	Total Dx	Δ	Tx1P	Tx2M	Tx2E	Tx2N	Total Tx	Δ		
Angola	130	130	27	21	130	70	21	6	21	16	21	1	130	70
Benin	3	3	0	0	3	0	0	0	0	0	0	0	3	0
Burkina Faso	8	8	2	2	8	0	2	2	2	0	2	0	8	0
Cameroon	13	10	9	6	14	0	11	0	7	8	11	1	14	0
Central African Republic	15	4	5	5	15	-4	10	3	3	7	10	-3	15	-4
Chad	52	52	7	7	52	-48	53	3	9	9	53	-47	53	-47
Congo	68	68	6	4	68	3	3	0	3	3	3	0	68	3
Côte d’Ivoire	29	29	3	2	29	25	2	2	2	2	2	1	29	25
Democratic Republic of the Congo	696	578	276	231	696	64	546	59	145	191	546	82	799	76
Equatorial Guinea	4	4	2	1	4	0	4	1	1	1	4	1	4	0
Gabon	5	5	1	1	5	0	1	0	1	1	1	0	6	0
Ghana	10	10	0	0	10	2	10	0	0	0	10	2	10	2
Guinea	125	125	3	3	125	10	3	2	0	3	3	0	125	10
Mali	7	7	3	0	8	0	0	0	0	0	0	0	8	0
Nigeria	50	50	5	5	50	0	5	0	0	5	5	0	50	0
South Sudan	60	59	6	5	60	45	5	5	5	5	5	0	60	45
Togo	2	2	0	0	2	0	0	0	0	0	0	0	2	0
Uganda	48	48	12	4	48	-86	4	4	4	4	4	0	48	-86
**Total**	1,325	1,192	367	297	1,327	81	680	87	203	255	680	38	1,432	94
**Δ**	83	143	13	25	81		40	4	-2	2	38		94	

DxC: clinical diagnosis; DxS: serological diagnosis; DxP: parasitological diagnosis; DxPh: disease staging. Tx1P: treatment of first-stage infection with pentamidine; Tx2M: treatment of second-stage infection with melarsoprol; Tx2E: treatment of second-stage infection with eflornithine; Tx2N: treatment of second-stage infection with nifurtimox-eflornithine combination therapy (NECT). Differences between the July 2019 survey and the June 2017 survey [2] in column ‘Δ’.

**Table 5:** Fixed health facilities for rhodesiense HAT: July 2019 survey. Differences between the July 2019 survey and the June 2017 survey [2] in column ‘Δ’.

Country	Diagnosis					Treatment				TOTAL	Δ
	DxC	DxP	DxPh	Total Dx	Δ	Tx1S	Tx2M	Total Tx	Δ		
Kenya	1	1	1	1	-24	1	1	1	-14	1	-24
Malawi	21	12	4	21	1	4	4	4	0	21	1
Rwanda	12	12	4	12	0	0	0	0	0	12	0
Uganda	38	37	12	38	1	10	10	10	0	38	1
United Republic of Tanzania	17	5	3	17	0	4	4	4	0	17	0
Zambia	16	16	16	16	4	14	14	14	4	16	4
Zimbabwe	6	6	6	6	5	6	1	6	5	6	5
**Total**	111	89	46	111	-13	39	34	39	-5	111	-13
**Δ**	-13	-5	-5	-13		-5	-10	-5		-13	

DxC: clinical diagnosis; DxP: parasitological diagnosis; DxPh: disease staging. Tx1S: treatment of first-stage infection with suramin; Tx2M: treatment of second-stage infection with melarsoprol. Differences between the July 2019 survey and the June 2017 survey [2] in column ‘Δ’.

For gambiense HAT, 1,432 fixed health facilities with capacity for diagnosis and/or treatment were listed (+7.0% since the June 2017 survey [2]). Diagnosis is available in 1,327 facilities (+6.5%) and treatment in 680 (+5.9%). More than half of the facilities (55.8%) are in the Democratic Republic of the Congo. For rhodesiense HAT, 111 facilities (-10.5%) offer diagnosis in seven endemic countries (i.e. Kenya, Malawi, Rwanda, Uganda, United Republic of Tanzania, Zambia and Zimbabwe) and treatment is provided in 39 health facilities.

The increase in gambiense HAT facilities was driven by the Democratic Republic of the Congo, which undertook a major redistribution of HAT capacities resulting in an overall increase of HAT-competent facilities (+76). In Angola (+70), and South Sudan (+45), the increase was related to the introduction of rapid tests in some endemic regions. By contrast, Chad and Uganda reduced the number of diagnostic facilities, a rebound that follows the large expansions undertaken in the previous five years [2, 10]. In particular, 47 and 86 sites with capacity for serological screening were discontinued in Chad and Uganda respectively. Among the criteria used to identify the sites to be discontinued was the absence of HAT detection in their catchment area. Sentinel sites remained active to cover these areas. In other gambiense HAT endemic countries little variation in competent facilities was reported.

Regarding rhodesiense HAT, the accessibility of diagnosis and treatment was increased in Zambia and Zimbabwe with 4 and 5 additional facilities respectively. These facilities with capacity for both HAT diagnosis and treatment were established in the transmission zones, where none previously existed. An overall reduction of the number of diagnostic facilities was reported in Kenya (-24), largely owing to retirement or transfer of skilled staff.

No specific health facilities able to diagnose and treat HAT are reported in Botswana, Burundi, Eswatini, Ethiopia, the Gambia, Guinea-Bissau, Liberia, Mozambique, Namibia, Niger, Senegal and Sierra Leone.

#### Population at risk potentially covered by fixed health facilities

The potential coverage of the population at risk of HAT by fixed health facilities is summarized in S3 File.

For gambiense HAT, approximately 31, 42, and 47 million people at risk are respectively within one-, three- and five-hour travel of a facility offering diagnosis. These values correspond to 60%, 82%, and 91% of the at-risk population in the period 2014–2018, and they mark a slight improvement over the 2012–2016 values when 58%, 79%, and 89% of the at-risk population was within one-, three- and five-hour travel [2]. For treatment, the population at risk within the same travel time thresholds is 23.5 million (46% of the at-risk population), 40 million (78%), and 46 million (89%), again only marginally better than in the 2012–2016 period.

For rhodesiense HAT the proportion of at risk population potentially covered by fixed health facilities is lower than for gambiense HAT. In particular, 35%, 67%, and 84% of the rhodesiense HAT at-risk population is within one-, three- and five-hour travel of a facility offering diagnosis, and for treatment the values are 27%, 58%, and 75%. These values are very close to those of the 2012–2016 period (i.e. 36%, 70%, and 85% for diagnosis and 24%, 63%, and 77% for treatment [2]).

### HAT elimination as a public health problem at country level

Using the indicator for elimination of HAT as public health problem at country level, as defined by the HAT-e-TAG, and the assessment of the intensity of control and surveillance activities, the eligibility of the different countries vis-à-vis the validation of HAT elimination as a public health problem was assessed.

Table 6 summarizes the outcome of this 2018 assessment for gambiense HAT endemic countries (Table 7 is for rhodesiense HAT endemic countries).

**Table 6:** Eligibility for validation of gambiense HAT elimination as a public health problem at the national level.

Country	<1 HAT case/10 000 inhabitants/year in all districts [2014–2018]	HAT surveillance and control activities	Eligibility
Angola	no	adequate	no
Benin	yes	adequate	yes
Burkina Faso	yes	adequate	yes
Cameroon	yes	adequate	yes
Central African Republic	no	insufficient	no
Chad	no	adequate	no
Congo	no	adequate	no
Côte d’Ivoire	yes	adequate	yes
Democratic Republic of the Congo	no	insufficient	no
Equatorial Guinea	no	adequate	no
Gabon	no	insufficient	no
Gambia	yes	absent	no
Ghana	yes	adequate	yes
Guinea	no	adequate	no
Guinea-Bissau	yes	absent	no
Liberia	yes	absent	no
Mali	yes	adequate	yes
Niger	yes	absent	no
Nigeria	yes	insufficient	no
Senegal	yes	absent	no
Sierra Leone	yes	absent	no
South Sudan	no	insufficient	no
Togo	yes	adequate	yes
Uganda	yes	adequate	yes

**Table 7:** Summary of the eligibility for validation of rhodesiense HAT elimination as a public health problem by country.

Country	<1 HAT case/10 000 inhabitants/year in all districts [2014–2018]	HAT Surveillance and control activities	Eligibility
Burundi	yes	absent	no
Ethiopia	yes	absent	no
Kenya	yes	insufficient	no
Malawi	no	adequate	no
Mozambique	yes	absent	no
Rwanda	yes	adequate	yes
Tanzania	yes	insufficient	no
Uganda	yes	insufficient	no
Zambia	yes	insufficient	no
Zimbabwe	yes	insufficient	no

* Botswana, Eswatini and Namibia are considered free of the vector for the transmission of *T*. *b*. *rhodesiense* HAT [23–26], and therefore different considerations should be made in assessing disease elimination.

Eight countries are considered to be eligible for validation of HAT elimination as a public health problem (i.e. Benin, Burkina Faso, Cameroon, Cote d’Ivoire, Ghana, Mali, Rwanda and Togo). Other countries fail to meet the criteria, either because of the number of cases by district (i.e. Angola, Chad, Congo, Equatorial Guinea, Guinea, Malawi and South Sudan), or because of the weakness of control and surveillance activities (i.e. Burundi, Ethiopia, Gambia, Guinea-Bissau, Kenya, Mozambique, Niger, Nigeria, Senegal, Sierra Leone, Tanzania, Zambia and Zimbabwe), or because of both (i.e. Central African Republic, Democratic Republic of the Congo, Gabon, South Sudan). In the case of Botswana, Eswatini and Namibia, despite the absence of medical activities, interventions against tsetse seem to have eliminated the vectors of HAT, and therefore different considerations should be made in assessing disease elimination. Uganda, being the only country reporting both gambiense and rhodesiense HAT, would be in the situation of meeting the criteria of elimination as public health for gambiense HAT but not for rhodesiense HAT, because of insufficient rhodesiense HAT control and surveillance activities.

## Discussion

The epidemiological trends reported in 2017–2018 are in line with those observed in previous years. The goal of elimination of HAT as public health problem is on track to be achieved, and the main global target for 2020 (i.e. < 2000 cases reported/year) has already been reached in 2017, with further progress in 2018. The attainment of this goal was considered by no means trivial when the target was set [29]. The figure of 977 cases reported in 2018 corresponds to a 96% decline compared to the year 2000. The reduction in the area estimated to be at moderate (or higher) risk of HAT (i.e. reporting ≥ 1 case/10,000 inhabitants/year) is also consistent with the progress in the control of the disease, and the corresponding 2020 target is close to being reached.

Concerning gambiense HAT, the data on reported number of cases are backed by strengthened active screening and a reinforcement of passive surveillance in the majority of endemic countries. Reported data are therefore believed to reflect a real abatement in disease transmission. In this context, eight of the 26 countries endemic for gambiense HAT have been identified as eligible to request validation of gambiense HAT elimination as a public health problem.

Despite this generally positive picture, areas where gambiense HAT is a public health issue still exist, and control and surveillance activities are still inadequate in a number of countries. Also, insecurity continues to hamper activities in some areas of Central African Republic, Democratic Republic of the Congo and South Sudan.

Reported cases of *T*. *b*. *rhodesiense* infection in humans have become increasingly scattered and sporadic, and they are mainly linked to spill over from the wildlife transmission cycle. Importantly, the level of under detection for rhodesiense HAT is believed to have increased. In particular, the widening use of rapid tests to diagnose malaria and the subsequent reduction in the use of blood smears is likely to have reduced accidental detection of HAT. The decreasing availability of microscopes, and a decline in the ability to use them, compounds the problem of rhodesiense HAT diagnosis. These problems are well documented in Malawi and Uganda, the countries reporting the highest number of rhodesiense HAT cases. Overall, control and surveillance of rhodesiense HAT have weakened, and the cases reported are likely to represent a small proportion of the real number of cases.

At this juncture in the process of elimination of HAT, Geographic Information Systems and the systematic mapping of epidemiological and control information are becoming increasingly critical to target activities. In particular, the Atlas of HAT [17] represents both the main tool to monitor progress towards the goals and a resource to support the planning of field operations. Furthermore, the Atlas is a unique source of data for a variety of modelling exercises [30–33].

In the present paper, the first assessment of eligibility for validation of HAT elimination at the national level is provided. To this end, a new indicator is presented, which is based on the number of reported cases by health district and the related population. The introduction of this new indicator was necessary because the 2020 global indicators and targets could not be directly used at, or adapted to, the country level. For example, it was not evident how the global target for annually-reported HAT cases (i.e. <2 000) could be broken down by country. Furthermore, the estimation of the area at risk, based on spatial smoothing [18], requires geospatial analysis tools and skills that are not readily available at the level of NSSCPs. Indeed, one of the main requirements for the national-level indicator was that it should be easy to calculate by NSSCPs, as these are in charge of developing and submitting the country validation dossiers to WHO. In this context, the choice of the health district as the basic geographical unit of analysis and reporting is in line with many other NTDs, including visceral leishmaniasis [34], trachoma [35] and lymphatic filariasis [36]; as such, it is expected to be easily managed by the NSSCPs.

## Conclusions

Beyond the 2020 goal of HAT elimination as a public health problem, which appears well within reach, the 2030 goal of elimination as interruption of HAT transmission must shift into focus. Through WHO coordination and with support from numerous stakeholders, the NSSCPs have played the key role in reaching the present point. This pivotal role of NSSCPs has to be sustained if past achievements are to be consolidated and the 2030 “zero cases” goal is to be reached. To this end, further efforts to increase awareness and ownership of elimination goals by national health authorities need to be made. Innovation in HAT control and surveillance is still needed, and intervention strategies need to be adapted to evolving epidemiological settings and to different countries and local contexts. In particular, integration of HAT activities in the regular health system is needed—a challenging endeavour for the weak health structures in many rural areas affected by HAT. Training for, and motivation of, health care staff also need to be sustained. Crucially, the continued commitment of resource partners needs to be ensured, so that the HAT elimination programme does not become victim of its own success.

Country-level validation of HAT elimination as public health problem needs to be pursued, so as to showcase past achievements and spur further progress. To this end, protocols and procedures have been developed, and a number of eligible countries have already started the process to obtain formal recognition from WHO.

Progress has been made in the development of new oral drugs capable of curing both stages of gambiense HAT, with clinical trials of fexinidazole successfully completed [37] leading to its introduction in the therapeutic protocols [38]; and beyond that, new perspectives could be opened by a single dose oral treatment (acoziborole [39]), currently under clinical evaluation.

At this important juncture, ensuring access to the best screening and diagnostic tools becomes a priority. Engagement of external funders and manufacturers is needed to guarantee their availability in the field, emulating the commitments to secure access to HAT treatment.

Existing and innovative tools and strategies for tsetse control have proved useful in decreasing vector abundance, and they contribute to curbing disease transmission when strategically deployed and coordinated with medical interventions [40].

The epidemiological role of asymptomatic human carriers and animal reservoirs of gambiense HAT remains unclear, and it requires further scrutiny [41]. Recent research strongly suggests that the skin may be a reservoir for trypanosomes [42]. Even though more research is needed, this finding could lead to the development of new diagnostic approaches.

As regards rhodesiense HAT, and taking into consideration its zoonotic nature, the interruption of its transmission poses major challenges. Addressing these challenges will only be possible in a “One health” framework. Indeed, the low burden of rhodesiense HAT makes it difficult to mobilize investment and to support research for improved control tools. In this context, ongoing efforts to improve the treatment of rhodesiense sleeping sickness [43] are both remarkable and commendable, especially taking into account the difficulty of running a clinical trial for a disease with a very low reported incidence.

## Supporting information

S1 File Area at risk of gambiense and rhodesiense HAT. Period 2014–2018 (by country).
(DOCX)

S2 File Population at risk of gambiense and rhodesiense HAT. Period 2014–2018 (by country).
(DOCX)

S3 File People at risk of HAT that are potentially covered by facilities with diagnostic and treatment capabilities for HAT. (DOCX)
