# Supplementary material for: Monitoring the elimination of human African trypanosomiasis at continental and country level: Update to 2018
Source: PLoS Negl Trop Dis. 2020 May 21;14(5):e0008261. doi: 10.1371/journal.pntd.0008261 (PMC7241700; doi:10.1371/journal.pntd.0008261)
Supplement: S1 File — Period 2014–2018 (by country). (DOCX) [file pntd.0008261.s001.docx]

# Area at risk of gambiense and rhodesiense HAT

Table 1 Areas at risk of *T. b. gambiense* infection (km^2^). Period 2014–2018.

| **Country** | **Total country area*** | **Area at risk**  **2014-2018** | | | | |
| --- | --- | --- | --- | --- | --- | --- |
|  |  | **Very High**  **and High** | **Moderate** | **Low and**  **Very Low** | **Total**  **at risk** | **% of total**  **country**  **area** |
| Angola | 1,253,770 | - | 1,682 | 72,172 | 73,853 | 5.9 |
| Burkina Faso | 274,470 | - | - | 1,241 | 1,241 | 0.5 |
| Cameroon | 466,396 | 185 | 1,162 | 7,318 | 8,665 | 1.9 |
| Central African Republic | 624,398 | 3,120 | 15,658 | 34,008 | 52,786 | 8.5 |
| Chad | 1,272,490 | 295 | 4,116 | 14,801 | 19,211 | 1.5 |
| Congo | 338,522 | 1,449 | 10,976 | 40,643 | 53,067 | 15.7 |
| Cote d'Ivôire | 321,363 | - | - | 4,154 | 4,154 | 1.3 |
| Democratic Republic of the Congo | 2,304,080 | 7,608 | 128,625 | 429,411 | 565,644 | 24.5 |
| Equatorial Guinea | 27,019 | - | 311 | 4,635 | 4,946 | 18.3 |
| Gabon | 265,978 | 607 | 4,794 | 6,176 | 11,577 | 4.4 |
| Guinea | 246,094 | 104 | 2,912 | 7,748 | 10,764 | 4.4 |
| Sierra Leone | 72,777 | - | 12 | 1,179 | 1,191 | 1.6 |
| South Sudan | 633,356 | - | 1,169 | 50,463 | 51,632 | 8.2 |
| Uganda | 205,540 | - | - | 7,666 | 7,666 | 3.7 |
| Other Endemic Countries** | 4,097,446 | - | - | - | - | - |
| Total | 12,403,699 | 13,367 | 171,416 | 681,614 | 866,397 | 7.0 |

* Land area. The area of surface water bodies as depicted in the Shuttle Radar Topography Mission—River-Surface Water Bodies dataset is not included.

** Countries at marginal risk: Benin, Gambia, Ghana, Guinea-Bissau, Liberia, Mali, Niger, Nigeria, Senegal and Togo.

Table 2 Areas at risk of *T. b. rhodesiense* infection (km^2^). Period 2014–2018.

| **Country** | **Total country area*** | **Area at risk**  **2014-2018** | | | | |
| --- | --- | --- | --- | --- | --- | --- |
|  |  | **Very High**  **and High** | **Moderate** | **Low and**  **Very Low** | **Total**  **at risk** | **% of total**  **country**  **area** |
| Malawi | 94,758 | - | 1,670 | 11,888 | 13,557 | 14.4 |
| United Republic of Tanzania | 886,278 | - | 1,284 | 10,916 | 12,201 | 1.4 |
| Uganda | 205,540 | - | - | 8,552 | 8,552 | 4.2 |
| Zambia | 742,479 | - | 6,690 | 24,997 | 31,687 | 4.3 |
| Zimbabwe | 388,414 | - | 277 | 5,187 | 5,464 | 1.4 |
| Other Endemic Countries** | 3,947,508 | - | - | - | - | - |
| Total | 6,264,977 | - | 9,922 | 61,541 | 71,462 | 1.1 |

* Land area. The area of surface water bodies as depicted in the Shuttle Radar Topography Mission—River-Surface Water Bodies dataset is not included.

** Countries at marginal risk: Botswana, Burundi, Ethiopia, Kenya, Mozambique, Namibia, Rwanda and Swaziland.
