# Supplementary material for: Monitoring the elimination of human African trypanosomiasis at continental and country level: Update to 2018
Source: PLoS Negl Trop Dis. 2020 May 21;14(5):e0008261. doi: 10.1371/journal.pntd.0008261 (PMC7241700; doi:10.1371/journal.pntd.0008261)
Supplement: S2 File — Period 2014–2018 (by country). (DOCX) [file pntd.0008261.s002.docx]

# Population at risk of gambiense and rhodesiense HAT

Table 1 Population at risk of *T. b. gambiense* infection (no. persons × 10^3^). Period 2014–2018.

| **Country** | **Total country population**  **2018*** | **Population at risk**  **2014-2018** | | | | | |
| --- | --- | --- | --- | --- | --- | --- | --- |
|  |  | **Very High**  **and High** | **Moderate** | **Low and**  **Very Low** | **Total**  **at risk** | **% of total**  **country**  **population** |  |
| Angola | 30,356 | - | 26 | 2,018 | 2,044 | 6.7 |  |
| Burkina Faso | 19,743 | - | - | 92 | 92 | 0.5 |  |
| Cameroon | 25,641 | - | 33 | 160 | 193 | 0.8 |  |
| Central African Republic | 5,745 | 34 | 162 | 543 | 740 | 12.9 |  |
| Chad | 15,833 | - | 112 | 651 | 763 | 4.8 |  |
| Congo | 5,062 | 15 | 49 | 2,474 | 2,538 | 50.1 |  |
| Cote d'Ivôire | 26,261 | - | - | 619 | 619 | 2.4 |  |
| Democratic Republic of the Congo | 85,181 | 98 | 4,930 | 32,463 | 37,490 | 44.0 |  |
| Equatorial Guinea | 797 | - | 10 | 41 | 51 | 6.4 |  |
| Gabon | 2,119 | 1 | 17 | 15 | 33 | 1.6 |  |
| Guinea | 11,855 | - | 149 | 2,869 | 3,017 | 25.5 |  |
| Sierra Leone | 6,312 | - | - | 178 | 179 | 2.8 |  |
| South Sudan | 10,205 | - | 44 | 1,977 | 2,021 | 19.8 |  |
| Uganda | 40,854 | - | - | 1,337 | 1,337 | 3.3 |  |
| Other Endemic Countries** | 313,125 | - | - | - | - | - |  |
| Total | 599,189 | 149 | 5,531 | 45,438 | 51,118 | 8.5 |  |

* As per Landscan

** Countries at marginal risk: Benin, Gambia, Ghana, Guinea-Bissau, Liberia, Mali, Niger, Nigeria, Senegal and Togo.

Table 2 Population at risk of *T. b. rhodesiense* infection (no. persons × 10^3^). Period 2014–2018.

| **Country** | **Total country population**  **2018*** | **Population at risk**  **2014-2018** | | | | |
| --- | --- | --- | --- | --- | --- | --- |
|  |  | **Very High**  **and High** | **Moderate** | **Low and**  **Very Low** | **Total**  **at risk** | **% of total**  **country**  **population** |
| Malawi | 19,843 | - | 56 | 978 | 1,035 | 5.2 |
| United Republic of Tanzania | 55,451 | - | 2 | 251 | 253 | 0.5 |
| Uganda | 40,854 | - | - | 1,060 | 1,060 | 2.6 |
| Zambia | 16,445 | - | 18 | 475 | 494 | 3.0 |
| Zimbabwe | 14,030 | - | 1 | 23 | 24 | 0.2 |
| Other Endemic Countries** | 213,919 | - | - | - | - | - |
| Total | 360,542 | - | 77 | 2,789 | 2,866 | 0.8 |

* As per Landscan

** Countries at marginal risk: Botswana, Burundi, Ethiopia, Kenya, Mozambique, Namibia, Rwanda and Swaziland.
