# Supplementary material for: Monitoring the elimination of human African trypanosomiasis at continental and country level: Update to 2018
Source: PLoS Negl Trop Dis. 2020 May 21;14(5):e0008261. doi: 10.1371/journal.pntd.0008261 (PMC7241700; doi:10.1371/journal.pntd.0008261)
Supplement: S3 File — (DOCX) [file pntd.0008261.s003.docx]

People at risk of HAT that are potentially covered by facilities with diagnostic and treatment capabilities for HAT.

Table 1 People at risk of HAT that are potentially covered by facilities with diagnostic and treatment capabilities for HAT

| **Risk category** | **People at risk** | **People at risk covered by facilities with HAT capabilities** | | | | | | | | | | | |
| --- | --- | --- | --- | --- | --- | --- | --- | --- | --- | --- | --- | --- | --- |
|  |  | **Diagnosis** | | | | | | **Treatment** | | | | | |
|  |  | **≤ 1-hour travel** | | **≤ 3-hour travel** | | **≤ 5-hour travel** | | **≤ 1-hour travel** | | **≤ 3-hour travel** | | **≤ 5-hour travel** | |
|  | (no. persons × 10^3^) | (no. persons × 10^3^) | % of at risk | (no. persons × 10^3^) | % of at risk | (no. persons × 10^3^) | % of at risk | (no. persons × 10^3^) | % of at risk | (no. persons × 10^3^) | % of at risk | (no. persons × 10^3^) | % of at risk |
| **gambiense HAT** | | | | | | | | | | | | | |
| High and very high | 149 | 104 | 70 | 130 | 87 | 136 | 91 | 100 | 67 | 131 | 88 | 137 | 92 |
| Moderate | 5,531 | 2,720 | 49 | 4,405 | 80 | 5,014 | 91 | 2,680 | 48 | 4,375 | 79 | 5,013 | 91 |
| Low and very low | 45,346 | 27,995 | 62 | 37,307 | 82 | 41,421 | 91 | 20,715 | 46 | 35,367 | 78 | 40,368 | 89 |
| Total | 51,118 | 30,819 | 60 | 41,842 | 82 | 46,571 | 91 | 23,495 | 46 | 39,873 | 78 | 45,518 | 89 |
| **rhodesiense HAT** | | | | | | | | | | | | | |
| High and very high | - | - | - | - | - | - | - | - | - | - | - | - | - |
| Moderate | 77 | 37 | 48 | 61 | 79 | 66 | 86 | 13 | 17 | 43 | 55 | 55 | 72 |
| Low and very low | 2,789 | 973 | 35 | 1,871 | 67 | 2,348 | 84 | 747 | 27 | 1,630 | 58 | 2,089 | 75 |
| Total | 2,866 | 1,010 | 35 | 1,932 | 67 | 2,414 | 84 | 760 | 27 | 1,672 | 58 | 2,144 | 75 |
